# Biosensing systems for the detection and quantification of methane gas

**DOI:** 10.1007/s00253-023-12629-7

**Published:** 2023-07-24

**Authors:** Noemi Poma, Andrea Bonini, Federico Vivaldi, Denise Biagini, Mariagrazia Di Luca, Daria Bottai, Fabio Di Francesco, Arianna Tavanti

**Affiliations:** 1 Department of Biology, University of Pisa, Via San Zeno 35-39, 56127 Pisa, Italy; 2 Present Address: Groningen Biomolecular Sciences and Biotechnology, University of Groningen, 9747 AG Groningen, The Netherlands; 3 Department of Chemistry and Industrial Chemistry, University of Pisa, Via Giuseppe Moruzzi 13, 56124 Pisa, Italy; 4 Metitech S.R.L., Via Livornese 835, 56122 Pisa, Italy

**Keywords:** Methanotrophs, Methane biosensing, Methane monooxygenase, Biosensors

## Abstract

**Abstract:**

Climate change due to the continuous increase in the release of green-house gasses associated with anthropogenic activity has made a significant impact on the sustainability of life on our planet. Methane (CH_4_) is a green-house gas whose concentrations in the atmosphere are on the rise. CH_4_ measurement is important for both the environment and the safety at the industrial and household level. Methanotrophs are distinguished for their unique characteristic of using CH_4_ as the sole source of carbon and energy, due to the presence of the methane monooxygenases that oxidize CH_4_ under ambient temperature conditions. This has attracted interest in the use of methanotrophs in biotechnological applications as well as in the development of biosensing systems for CH_4_ quantification and monitoring. Biosensing systems using methanotrophs rely on the use of whole microbial cells that oxidize CH_4_ in presence of O_2_, so that the CH_4_ concentration is determined in an indirect manner by measuring the decrease of O_2_ level in the system. Although several biological properties of methanotrophic microorganisms still need to be characterized, different studies have demonstrated the feasibility of the use of methanotrophs in CH_4_ measurement. This review summarizes the contributions in methane biosensing systems and presents a prospective of the valid use of methanotrophs in this field.

**Key points:**

*•* *Methanotroph environmental relevance in methane oxidation*

*•* *Methanotroph biotechnological application in the field of biosensing*

*•* *Methane monooxygenase as a feasible biorecognition element in biosensors*

## Introduction

Methane (CH_4_) is an odorless and colorless gas widely used in the generation of electricity and as a heating fuel. It is an important green-house gas (GHG) being the second biggest contributor to global warming, with a global warming potential 28 times higher than carbon dioxide (CO_2_) over a 100-year period since it is a stronger absorber of the infrared radiation with a thermal effect (wavelength 2.5-15 µm) that is emitted by the Earth when exposed to temperatures above 0 °C (Myhre et al. 2013). CH_4_ concentration in the atmosphere has shown a continuous increase, from an estimated value of around 700 ppb prior to the industrial revolution up to 1895.7 ppb in 2021 (Etheridge et al. 1998; NOAA 2022). Such increase is mostly attributed to anthropogenic activities, as it is estimated that more than 50% of CH_4_ emissions in the atmosphere are related to industrial activities, livestock farming, agriculture, the use of fossil fuels, biomass burning, rice paddies, among others (Saunois et al. 2020; IPCC 2021; EEA 2022; Zhang et al. 2022).

CH_4_ sinks are determinant since they contribute to the decrease CH_4_ levels in the atmosphere. Hydroxyl (OH) and chlorine (Cl) radicals constitute the primary sink, followed by microbial oxidation in soils by methanotrophic microorganisms (Saunois et al. 2020; Jackson et al. 2021). Methanotrophs are prokaryotes recognized for their unique capacity to utilize CH_4_ as a sole source of carbon and energy and are known to play a crucial role in the Earth’s biogeochemical carbon cycle (Hanson and Hanson 1996). They are widely distributed in the environment and have an important role in the consumption of atmospheric CH_4_, as well as in the capture of CH_4_ generated biologically or geothermally before it is released in the environment, acting as biofilters (Kalyuzhnaya et al. 2019). It is estimated that aerobic methanotrophs in upland soils consume up to 30 Tg CH_4_ per year, which corresponds to 6% of the global methane sinking capability (Shukla et al. 2013). The ability of methanotrophs to oxidize CH_4_ at ambient temperature has attracted increasing attention because of their potential use in bioremediation strategies, biotechnological applications, and the development of biosensing systems (Strong et al. 2015; Kwon et al. 2019; Gęsicka et al. 2021; Guerrero-Cruz et al. 2021). Particularly, CH_4_ detection and monitoring is important for both the environmental health and the human safety in domestic and industrial settings (Lawrence 2006; Aldhafeeri et al. 2020). Leaking of natural gas, which is comprised of 95% of CH_4_, is an important issue, since it is estimated that each year, just in the United States, around 9 million of tons of natural gas leak in the atmosphere during extraction transport and storage, directly contributing to the increase of CH_4_ concentration. Moreover, CH_4_ is highly flammable and forms explosive mixtures in the range of 5–15% v/v in air; indeed, accidental leaks have led to the occurrence of important explosions. Besides the risk associated to its flammability, inhalation of CH_4_ can cause suffocation and can be fatal if the levels of oxygen (O_2_) are lower that 12% (Lawrence 2006; Patel 2017).

Different analytical methods to detect CH_4_ are available; among them are gas chromatography (GC), select ion flow tube-mass spectrometry (SIFT-MS), infrared (IR) spectroscopy, and electrochemistry. Each of these techniques has its own advantages and disadvantages. For example, GC is widely used in gas detection, and it has a low LOD being suitable to detect small leaks of CH_4_; however, it needs cumbersome and costly instrumentation, making it very difficult to perform measurements outside of a laboratory. SIFT-MS is instead a portable system with the potential to be used in different environments; it has been recently used for the breath analysis; however, the sensitivity and specificity are issues that still need to be optimized (Langford 2023). IR spectroscopy allows a selective and specific detection of gases; however, it is a laboratory-based technique, requires qualified personnel, and has high running costs (Kamieniak et al. 2015). Due to the constraints of the classical methods, new reliable and cost-effective techniques should be developed. In this context, sensors have achieved great notoriety and have proven to be useful from environmental to clinical applications (Bonini et al. 2020, 2021; Vivaldi et al. 2020; Poma et al. 2021). Sensing systems for CH_4_ measurement using optical, calorimetric, pyroelectric, and electrochemical transduction methods have been described in literature. A comparison of the different detection methods in CH_4_ sensing has been provided by Aldhafeeri and colleagues (Aldhafeeri et al. 2020), where the advantages and disadvantages have been clearly presented. For example, optical detection sensing methods are non-destructive and able to operate without oxygen, but these are costly, have a high-power consumption, and have some selectivity issues. Instead, devices using calorimetry as transduction method have good selectivity and simplistic design and are easy to manufacture; however, these require harsh operating conditions and have a short life span and low detection accuracy. The characteristics of  electrochemical sensors like  their low-cost, high sensitivity, ease of use, portability, ease of miniaturization and the possibility to perform a remote monitoring made them a valid alternative in the control of generated greenhouse gasses at their source (Lawrence 2006; Kamieniak et al. 2015). Electrochemical CH_4_ detection can be achieved through its direct oxidation on the electrode surface; however, this approach needs aprotic solvents and specific electrolytes. Alternatively, the direct adsorption of CH_4_ onto a noble metal (e.g., platinum) electrode surface can be employed. Semi-conductive metals oxides like tin oxide are also used to produce solid state sensors, but these systems mostly operate at high temperatures (above 400 °C) to oxidize CH_4_ and suffer from poor selectivity, even though the use of filters containing noble metals catalysts and dopants can reduce this problem (Lawrence 2006; Sekhar et al. 2016). In this scenario, methanotrophs able to oxidize CH_4_ at ambient temperature and pressure may represent a valid alternative for the development of innovative CH_4_ biosensing systems using the whole cells or the enzymes participating in CH_4_ oxidation. Considering as well the variety of environments where methanotrophs can be isolated, the sensing devices could be suited to different applications. This minireview provides an overview on biosensing systems using methanotrophic bacteria in combination with electrochemical transduction techniques, focusing on the characteristics of methanotrophs as well as on the description of biosensing systems so far developed for CH_4_ detection, quantification, and monitoring.

### Methanotrophic microorganisms

Methanotrophs are microorganisms able to oxidize CH_4_ under aerobic or anaerobic conditions using different electron acceptors. They are ubiquitous in the environment, as their presence has been demonstrated in a variety of terrestrial and aquatic ecosystems (e.g., soil, mud, rivers, sediments, and sewage water), including extreme environments (e.g., hot springs, alkaline lakes, and permafrost) (Trotsenko and Khmelenina 2002; Semrau and DiSpirito 2019; Houghton et al. 2019; Guerrero-Cruz et al. 2021). Methanotrophs described up to date belong to the phyla *Proteobacteria*, *Verrucomicrobia*, and NC10. In addition, members of the Archaea domain have also been found (Kalyuzhnaya et al. 2019; Guerrero-Cruz et al. 2021).

Aerobic methanotrophs use oxygen as the electron acceptor during the oxidation of CH_4_ to methanol as shown in reaction (1):

1 $${\mathrm{CH}}_{4}+{\mathrm{O}}_{2}+\mathrm{NADH}+{\mathrm{H}}^{+}\to {\mathrm{CH}}_{3}\mathrm{OH}+{\mathrm{H}}_{2}\mathrm{O}+{\mathrm{NAD}}^{+}$$

On the contrary, in anaerobic methanotrophic archaea sulfate is used as the final electron acceptor (Knittel and Boetius 2009; Bhattarai et al. 2019), according to the reaction (2):

2 $${\mathrm{CH}}_{4}+{\mathrm{SO}}_{4}^{2-}\to {\mathrm{HCO}}_{3}^{-}+{\mathrm{HS}}^{-}+{\mathrm{H}}_{2}\mathrm{O}$$

In aerobic methanotrophs, the CH_4_ oxidation is catalyzed by the metalloenzymes, known as methane monooxygenases (MMOs), able to break the strong C-H bond (∆H = 105 kcal/mol) (Banerjee et al. 2019). The methyl-coenzyme M reductase is thought to be involved in the oxidation of CH_4_ in anaerobic conditions (Bhattarai et al. 2019; Thauer 2019).

### Methane monooxygenases (MMOs)

**Fig. 1:**
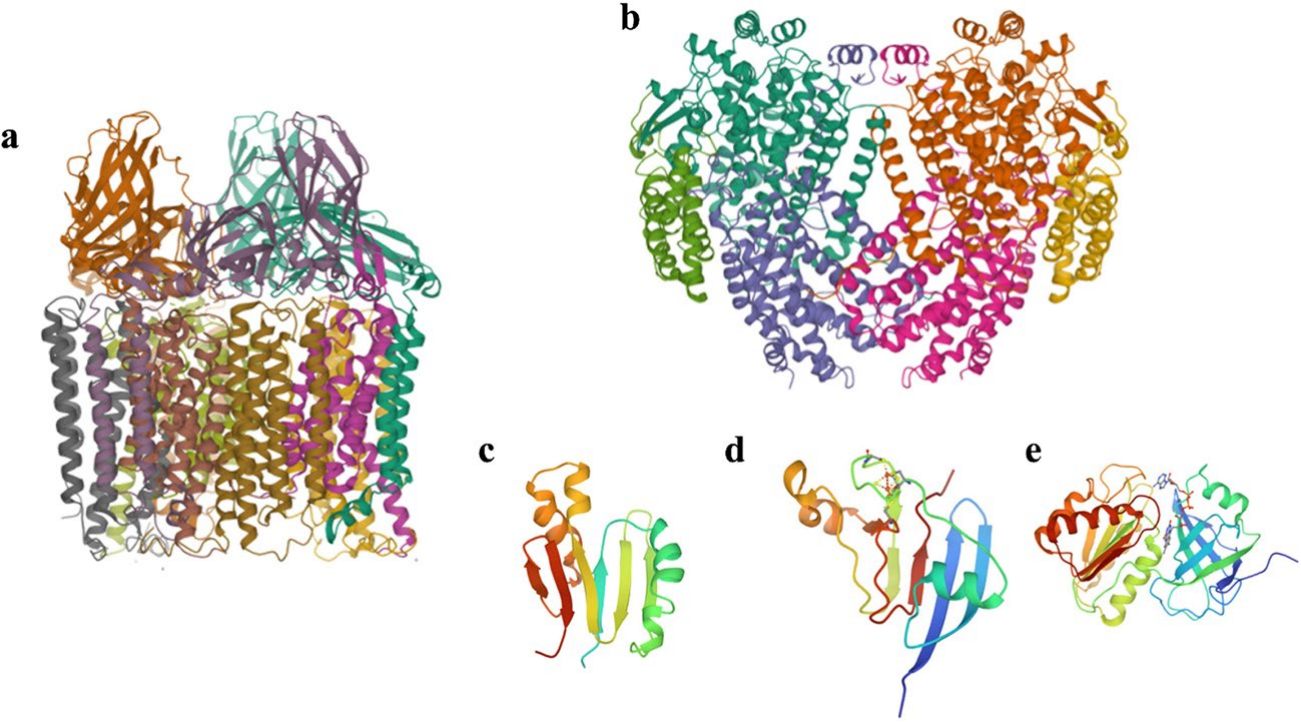
Structures of pMMO and sMMO components. X-ray crystal structures of (**a**) pMMO (PDB: 3RGB), (**b**) X-ray crystal structure of MMOH (PDB: 1MTY). NMR structures of (**c**) MMOB (PDB: 2MOB), (**d**) [2Fe-2S] domain of MMOR (PDB: 1JQ4), and (**e**) FAD and NADH binding domain of MMOR (PDB: 1TVC)

In aerobic methanotrophs, two types of MMOs are known, i.e., the membrane-bound particulate methane monooxygenase (pMMO) and the cytoplasmatic enzyme soluble methane monooxygenase (sMMO) (Ross and Rosenzweig 2017). Nearly all methanotrophs possess the pMMO enzyme, while sMMO is only present in just a few methanotrophs such as *Methylococcus capsulatus* strain Bath, and other bacterial species belonging to the genera *Methylosinus*, *Methylocystis*, *Methylomicrobium*, and *Methylomonas* (Murreil et al. 2000; Khider et al. 2021). Both sMMO and pMMO oxidase CH_4_, but they are different in structure, active site composition, and substrate selectivity (Sirajuddin and Rosenzweig 2015; Chan et al. 2021). The enzyme pMMO is a copper-dependent protein including only one hydroxylase component, which is a trimer of around 300 kDa containing three subunits PmoA, PmoB, and PmoC (Fig. 1a) (Khider et al. 2021). pMMO can also oxidase *n*-alkanes and *n-*alkenes (Sirajuddin and Rosenzweig 2015). The difficulties associated with the isolation and solubilization of the membrane enzyme pMMO have led to a better characterization of the less prevalent enzyme sMMO; these studies have mostly focused on two bacterial species, namely, *Methylococcus capsulatus* (Bath) and *Methylosinus trichosporium* OB3b. The catalytic activity of sMMO is achieved by three components, the 245 kDa hydroxylase (MMOH), a 16 kDa regulatory protein (MMOB), and the 40 kDa reductase (MMOR) (Fig. 1b–e) (Banerjee et al. 2019). MMOH is an homodimeric protein composed of three subunits (α, β, and γ), with a diiron active site present in the α subunit. MMOR is a nicotinamide adenine dinucleotide (NADH)-dependent protein containing flavin adenine dinucleotide (FAD) and an iron sulfur [2Fe-2S] domain, which delivers two electrons to the active center of MMOH through the oxidation of NADH (Ross and Rosenzweig 2017; Banerjee et al. 2019). MMOB forms a complex with MMOH, and its presence is known to increase the catalytic activity (Banerjee et al. 2019). sMMO has a wider substrate range including *n*-alkanes, *n*-alkenes, aromatic, and heterocyclic compounds, but only CH_4_ is relevant for the cell metabolism (Banerjee et al. 2019; Khider et al. 2021). sMMO is encoded by an operon composed of six genes, namely *mmoXYZBCD*, *mmoX*, *mmoY*, *mmoZ*, codifying for the α, β, and γ subunit of MMOH, respectively, while MMOB and MMOR are encoded by *mmoB* and *mmoC*, respectively. An additional gene *mmoD* is predicted to encode for a regulatory protein (Khider et al. 2021).

## The use of biosensing systems in methane quantification

**Fig. 2:**
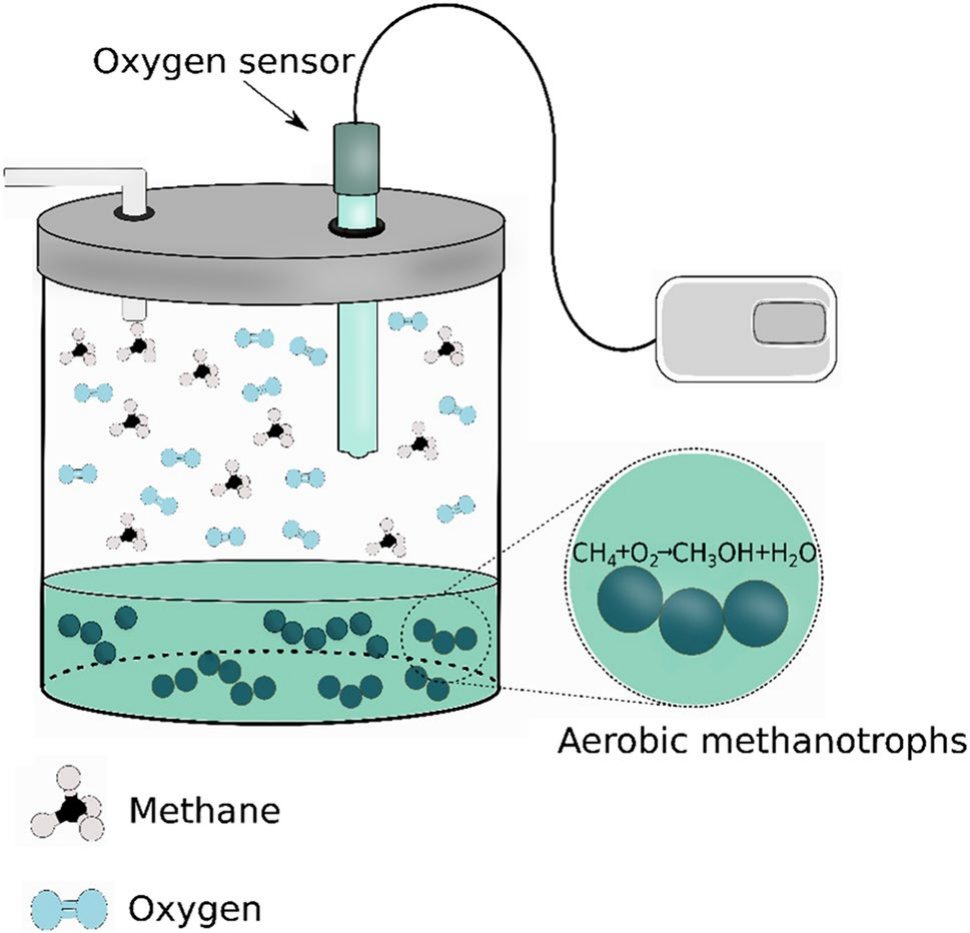
Working principle of methane biosensing systems

The peculiar ability of methanotrophs to oxidize CH_4_ has attracted the attention toward their use in the development of biosensing systems for its quantification and monitoring. Bacterial cells, known to use CH_4_ as a carbon source (Table 1), have been mainly used in combination with electrochemical sensors to measure O_2_ consumption resulting from the oxidation of CH_4_ (Fig. 2).

**Table 1:** Analytical figures of merit and operating conditions relevant to biosensing systems for CH_4_ detection and quantification

Microorganism/biorecognition element	Response time (s)*	Linear range	Detection limit	RSD (%)	Lifetime	Operating conditions (°C)^**^	Ref
*Methylomonas flagellata*	60	0–6.6 mM	13.1 μM	5	20 days/500 assays	30	(Okada et al. 1981)
*Methylomonas flagellata*	60	0–6.6 mM	5 μM	5	10 days/250 assays	30	(Karube et al. 1982)
*Pseudomonas aeruginosa* strain M16 and *Klebsiella* sp. strain M17	100	0.4–2.2 mM	0.1 mM	3	9 h/36 assays	20–25	(Wen et al. 2008)
*Klebsiella* sp. strain M17	< 90	0-7.1 mM	88 µM	3.5	12 h/50 assays	25	(Zhao et al. 2009)
*Methylosinus trichosporium* OB3b	20–100	0– 40 mM *P*CH_4_	N.D	N.D	Months	10–30	(Damgaard and Revsbech 1997)
*Methylosinus trichosporium* OB3b	60	0–10 mM *P*CH_4_	N.D	N.D	Months	21	(Damgaard et al. 1998)

*PCH*_*4*_, CH_4_ partial pressure; *RSD*, relative standard deviation; *N.D.*, not determined.

* Time required to obtain a steady-state signal of 95% after being exposed to the analyte^**^Optimal operating conditions

**Fig. 3:**
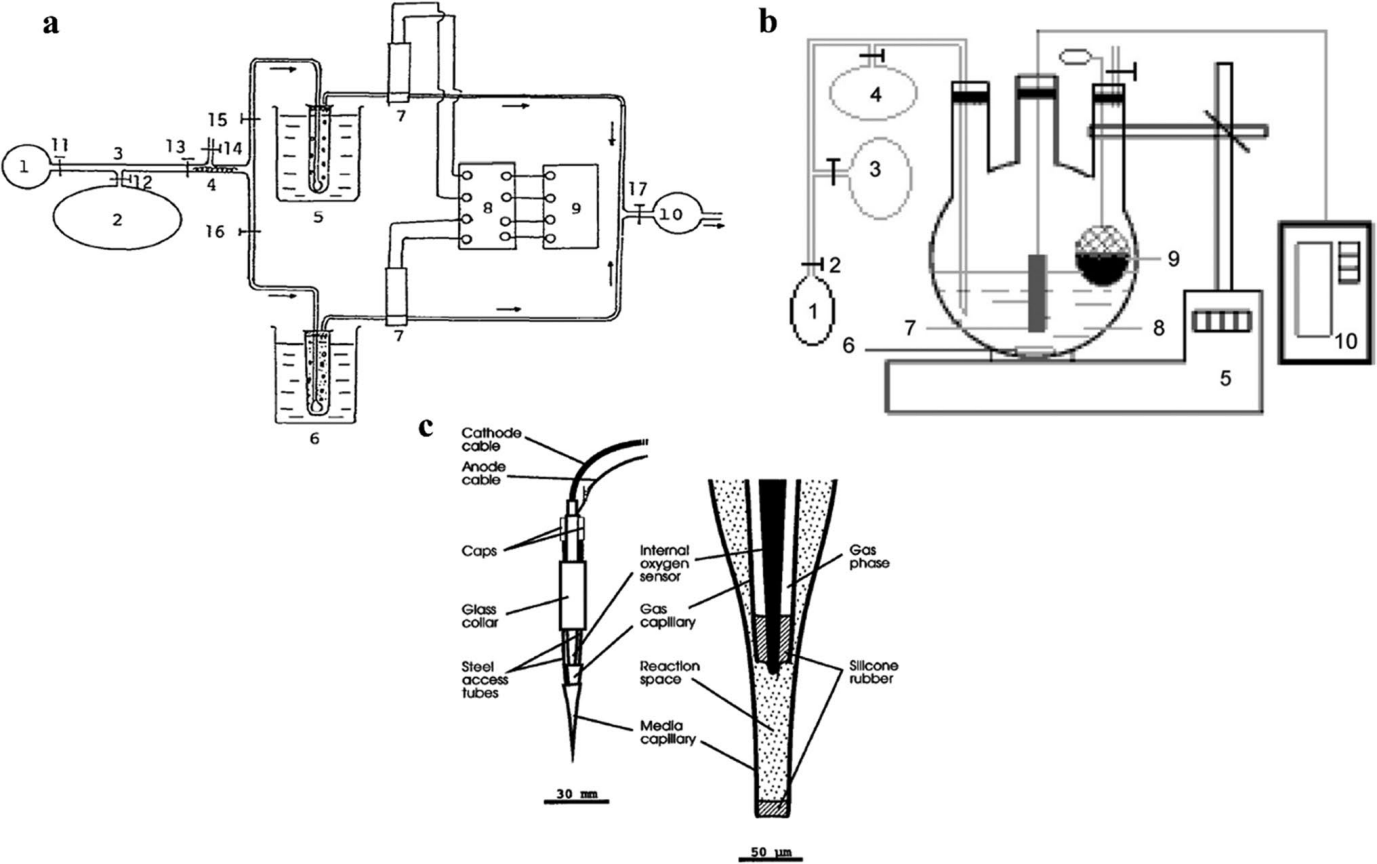
Schematic representation of CH_4_ biosensing systems. (**a**) Sensing system based on the use of CH_4_ oxidizing bacteria: 1, vacuum pump; 2, sample gas bag; 3, gas sample line; 4, cotton filter; 5, control reactor; 6, reactor containing *M. flagellata*; 7, O_2_ electrode; 8, amplifier; 9, recorder; 10, vacuum pump; 11–17, glass stopcocks (Okada et al. 1981; Karube et al. 1982) (reprinted from (Karube et al. 1982) with permission from Elsevier). (**b**) Sensing system for CH_4_ measurement in solution: 1, pump; 2, gas valve; 3, sample gas; 4, flow-meter; 5, thermostat magnetic stirrer; 6, magnetic bar; 7, oxygen sensor; 8, phosphate buffer solution; 9, bacterial beads; and 10, datalogger and computer (Wen et al. 2008; Zhao et al. 2009) (reprinted from (Wen et al. 2008) with permission from Elsevier). (**c**) Entire system (left) microsensor tip (right), composed of a gas and a media capillary. An internal O_2_ electrode is present in the gas capillary which serves as the O_2_ reservoir (Damgaard and Revsbech 1997) (reprinted from (Damgaard and Revsbech 1997) with permission from ACS)

The first works, by Okada et al. 1981 (Okada et al. 1981) and Karube et al. 1982 (Karube et al. 1982), depicted a system composed of two reactors, two oxygen electrodes, and a series of valves and tubes allowing the influx of CH_4_ and air into the reactors and toward the O_2_ electrodes under the control of a pump (Fig. 3a). In such systems, only one of the reactors contained the methanotrophic bacteria *Methylomonas flagellata*, so the difference in the current signal between the O_2_ electrodes was registered and used as the analytical signal. The decrease in the O_2_ concentration was directly associated to the CH_4_ oxidation and shown to be dependent on its concentration. Okada and collaborators (Okada et al. 1981) used *M. flagellata* bacterial cells entrapped on acetylcellulose filters coated with agar. The system showed a linear response below 6.6 mM, with a limit of detection (LOD) of 13.1 μM. Karube and collaborators (Karube et al. 1982) used instead *M. flagellata* microbial cells in suspension, obtaining a linear response below 6.6 mM and an LOD of 5 μM. The response of both systems was compared with measurements performed by gas chromatography with a thermal conductivity detector, and a significant correlation was established. However, none of these systems was able to provide a continuous measurement, since oxygen had to be introduced for the measurement and then restored. Subsequent studies by Wen et al. (Wen et al. 2008) and Zhao et al. (Zhao et al. 2009) similarly reported the development of measuring systems based on the use of reactors to quantify the CH_4_ concentration in the aqueous phase by using electrodes measuring dissolved O_2_ (Fig. 3b). The reactors contained bacterial strains of *Pseudomonas aeruginosa* (ME16) and *Klebsiella* sp. (ME17), both capable of oxidizing CH_4_, which were co-immobilized in polyvinyl alcohol (PVA), alginate, and boric acid beads. This system showed a linear response in the range of 0.4–2.2 mM, with an LOD of 0.1 mM (Wen et al. 2008). Differently, Zhao et al. only used the *Klebsiella* sp. strain ME17 immobilized in polyvinyl alcohol (PVA)-boric acid beads. In this work, a linear response from 0-7.1 mM CH_4_, with an LOD of 88 µM was reported (Zhao et al. 2009). Although these early sensing systems did not allow for an in situ monitoring and continuous measurement, they proved it feasible to measure CH_4_ using whole bacterial cells.

Systems for the continuous measurement of CH_4_ have also been described, for example, from Damgaard and colleagues (Damgaard and Revsbech 1997) who developed a microscale sensor composed of an O_2_ microsensor and two glass capillaries. One gas capillary served as the O_2_ reservoir and housed the microsensor, whose tip protruded in the reaction space within the media capillary that contained the bacterial cells of *M. trichosporium* OB3b (Fig. 3c). This system was based on the counter diffusion principle, i.e., in the presence of CH_4_ the bacterial cells consumed the O_2_ from the reservoir and the decrease in O_2_ concentration was registered by the microsensor; a linear response was obtained range from 0–40 mM of CH_4_ partial pressure, with a response time of 20–100 s. However, O_2_ diffusing from the environment may act as a major interferent, so this device would be useful only under anoxic conditions. This system was also tested in presence of other compounds such as H_2_S, CH_3_COOH, NH_3_, CO_2_ and H_2_, among them only H_2_S was determined to be an interferent. In 2001, this microsensor was applied to the measurement of CH_4_ microprofile in a sewage outlet biofilm, a system where both methanogenesis and methane oxidation occur (Damgaard et al. 2001). In another work, the same authors showed a modification of the aforementioned device by the addition of an external capillary where heterotrophic bacteria such as *Agrobacterium radiobacter* were introduced. Under those conditions, the O_2_ present in the environment would be first consumed, avoiding its entrance to the gas capillary and consequently the O_2_ interference to the signal. A linear range of 0–10 mM CH_4_ partial pressure was obtained, with a response time of 60 s. This system was applied to the measurement of a CH_4_ microprofile in a rice paddy soil (Damgaard et al. 1998).

So far, only one study described a sensor using the sMMO enzyme as a biorecognition element instead of the whole cell for the detection of CH_4_ (Chuang and Engineering 2005). In this proof-of-concept study, the MMOH component from *M. capsulatus* was immobilized onto a gold electrode previously modified with an oligopeptide. Throughout the application of a potential, electrons were transferred from the electrode to the enzyme catalytic site. Cyclic voltammetry was employed as transduction technique, and acetonitrile (CH_3_CN) was first used as a substrate to validate the system. An increase in the registered current signal was observed in the presence of increased CH_3_CN concentrations, and most of this signal was attributed to the MMOH on the electrode. In the presence of different CH_4_ concentrations, a response was recorded and associated to the presence of the enzyme. However, in contrast to CH_3_CN, when CH_4_ was used, an inconsistent response was registered, which could be attributed to the kinetics of CH_4_ partitioning between gas and liquid phase. Besides of these results, this work showed the practicability of this strategy and demonstrated the feasibility of the direct CH_4_ measurement.

## Conclusions

The development of methane sensing systems is relevant to the industrial and household safety and also to the environment protection. The unique characteristic of methanotrophs using CH_4_ as the sole source of carbon and energy, due to the presence of MMOs in aerobic environments, has inspired the development of CH_4_ biosensing systems. So far, just a few studies of this type are available; in such works, an indirect determination of CH_4_ is mainly performed by using whole bacterial cells and sensors measuring the O_2_ concentration. These pioneering studies proved the use of bioreactors containing methanotrophs feasible, while later studies progressed toward the development of microsensors performing continuous measurements and characterizing the CH_4_ microprofile in samples such as lake sediments, rice paddies, and biofilms. The use of the enzyme sMMO as a biorecognition element in the construction of a biosensor for CH_4_ was depicted in a proof-of-concept study. This work demonstrated the possibility of using this enzyme in the CH_4_ measurement and paves the way for future applications of MMOs in the set-up of methane sensing devices. The increasing interest in the use of methanotrophs in different biotechnological applications has led to a considerable progress in the understanding of methanotrophs biology. Particularly, the acquired knowledge of the structure and function of MMOs, together with the use of new available strategies for their modification and their heterologous expression, may help in their implementation as biorecognition elements in the development of enzymatic biosensors. There is still work to be done, but we believe that such systems soon could be used.

## References

[CR1] Aldhafeeri T, Tran MK, Vrolyk R, Pope M, Fowler M (2020). A review of methane gas detection sensors: recent developments and future perspectives. Inventions.

[CR2] Banerjee R, Jones JC, Lipscomb JD (2019). Soluble methane monooxygenase. Annu Rev Biochem.

[CR3] Bhattarai S, Cassarini C, Lens PNL (2019). Physiology and distribution of archaeal methanotrophs that couple anaerobic oxidation of methane with sulfate reduction. Microbiol Mol Biol Rev.

[CR4] Bonini A, Di Francesco F, Salvo P, Vivaldi F, Herrera E, Melai B, Kirchhain A, Poma N, Mattonai M, Caprioli R, Lomonaco T (2020) A graphenic biosensor for real-time monitoring of urea during dialysis. IEEE Sens J 1–1. 10.1109/jsen.2020.2966456

[CR5] Bonini A, Poma N, Vivaldi F, Biagini D, Bottai D, Tavanti A, Di Francesco F (2021) A label-free impedance biosensing assay based on CRISPR/Cas12a collateral activity for bacterial DNA detection. J Pharm Biomed Anal 204. 10.1016/j.jpba.2021.114268 10.1016/j.jpba.2021.114268 34298471

[CR6] Chan SI, Chang WH, Huang SH, Lin HH, Yu SSF (2021). Catalytic machinery of methane oxidation in particulate methane monooxygenase (pMMO). J Inorg Biochem.

[CR7] Chuang JD (2005) Electrochemistry of soluble methane monooxygenase on a modified gold electrode: implications for chemical sensing in natural waters. Master's thesis, Massachusetts Institute of Technology. https://dspace.mit.edu/handle/1721.1/31156

[CR8] Damgaard LR, Revsbech NP (1997). A microscale biosensor for methane containing methanotrophic bacteria and an internal oxygen reservoir. Anal Chem.

[CR9] Damgaard LR, Revsbech NP, Reichardt W (1998). Use of an oxygen-insensitive microscale biosensor for methane to measure methane concentration profiles in a rice paddy. Appl Environ Microbiol.

[CR10] Damgaard LR, Nielsen LP, Revsbech NP (2001). Methane microprofiles in a sewage biofilm determined with a microscale biosensor. Water Res.

[CR11] Etheridge DM, Steele LP, Francey RJ, Langenfelds RL (1998). Atmospheric methane between 1000 A.D. and present: evidence of anthropogenic emissions and climatic variability. J Geophys Res Atmos.

[CR12] European Environmental Agency (2022) Methane emissions in the EU: the key to immediate action on climate change. https://www.eea.europa.eu/publications/methane-emissions-in-the-eu. Accessed 26 Feb 2023

[CR13] Gęsicka A, Oleskowicz-Popiel P, Łężyk M (2021). Recent trends in methane to bioproduct conversion by methanotrophs. Biotechnol Adv.

[CR14] Guerrero-Cruz S, Vaksmaa A, Horn MA, Niemann H, Pijuan M, Ho A (2021) Methanotrophs: discoveries, environmental relevance, and a perspective on current and future applications. Front Microbiol 12. 10.3389/fmicb.2021.678057 10.3389/fmicb.2021.678057 PMC8163242 34054786

[CR15] Hanson RS, Hanson TE (1996). Methanotrophic bacteria. Microbiol Rev.

[CR16] Houghton KM, Carere CR, Stott MB, McDonald IR (2019). Thermophilic methanotrophs: in hot pursuit. FEMS Microbiol Ecol.

[CR17] Jackson RB, Abernethy S, Canadell JG, Cargnello M, Davis SJ, Féron S, Fuss S, Heyer AJ, Hong C, Jones CD, Damon Matthews H, O’Connor FM, Pisciotta M, Rhoda HM, de Richter R, Solomon EI, Wilcox JL, Zickfeld K (2021). Atmospheric methane removal: a research agenda. Philos Trans R Soc A Math Phys Eng Sci.

[CR18] Kalyuzhnaya MG, Gomez OA, Murrell JC (2019) The methane-oxidizing bacteria (methanotrophs). In: Taxonomy, Genomics and Ecophysiology of Hydrocarbon-Degrading Microbes. pp 1–34

[CR19] Kamieniak J, Randviir EP, Banks CE (2015). The latest developments in the analytical sensing of methane. TrAC - Trends Anal Chem.

[CR20] Karube I, Okada T, Suzuki S (1982). A methane gas sensor based on oxidizing bacteria. Anal Chim Acta.

[CR21] Khider MLK, Brautaset T, Irla M (2021) Methane monooxygenases: central enzymes in methanotrophy with promising biotechnological applications. World J Microbiol Biotechnol 37. 10.1007/s11274-021-03038-x 10.1007/s11274-021-03038-x PMC7994243 33765207

[CR22] Knittel K, Boetius A (2009). Anaerobic oxidation of methane: progress with an unknown process. Annu Rev Microbiol.

[CR23] Kwon M, Ho A, Yoon S (2019). Novel approaches and reasons to isolate methanotrophic bacteria with biotechnological potentials: recent achievements and perspectives. Appl Microbiol Biotechnol.

[CR24] Langford VS (2023) SIFT-MS: quantifying the volatiles you smell…and the toxics you don’t. Chemosensors 11(2):111. 10.3390/chemosensors11020111

[CR25] Lawrence NS (2006). Analytical detection methodologies for methane and related hydrocarbons. Talanta.

[CR26] IPCC (2021) Climate change 2021: the physical science basis. In: Masson-Delmotte V, Zhai P, Pirani A, Connors SL, Péan C, Berger S, Caud N, Chen Y, Goldfarb L, Gomis MI, Huang M, Leitzell K, Lonnoy E, Matthews JBR, Maycock TK, Waterfield T, Yelekçi O, Yu R, Zhou B (eds) Cambridge: Cambridge University Press, pp 2391. 10.1017/9781009157896

[CR27] Murreil JC, Gilbert B, McDonald IR (2000). Molecular biology and regulation of methane monooxygenase. Arch Microbiol.

[CR28] Myhre G, Shindell D, Bréon F-M, Collins W, Fuglestvedt J, Huang J, Koch D, Lamarque J-F, Lee D, Mendoza B, Nakajima T, Robock A, Stephens G, Takemura T, Zhang H (2013) IPCC Fifth Assessment Report (AR5) Chapter 8: Anthropogenic and Natural Radiative Forcing. In: Stocker TFD, Qin G-K, Plattner M, Tignor SK, J. A, Boschung A, Nauels Y, Xia VB, Midgley PM (eds) Climate change 2013: the physical science basis. Contribution of Working Group I to the Fifth Assessment Report of the Intergovernmental Panel on Climate Change. Cambridge University Press, Cambridge, United Kingdom and New York, NY, USA

[CR29] NOAA (2022) Increase in atmospheric methane set another record during 2021. https://www.noaa.gov/news-release/increase-in-atmospheric-methane-set-another-record-during-2021. Accessed 27 Feb 2023

[CR30] Okada T, Karube I, Suzuki S (1981). Microbial sensor system which uses *Methylomonas* sp. for the determination of methane. Eur J Appl Microbiol Biotechnol.

[CR31] Patel P (2017). Monitoring Methane. ACS Cent Sci.

[CR32] Poma N, Vivaldi F, Bonini A, Salvo P, Kirchhain A, Ates Z, Melai B, Bottai D, Tavanti A, Di Francesco F (2021) Microbial biofilm monitoring by electrochemical transduction methods. TrAC - Trends Anal Chem 134. 10.1016/j.trac.2020.116134

[CR33] Ross MO, Rosenzweig AC (2017). A tale of two methane monooxygenases. J Biol Inorg Chem.

[CR34] Saunois M, Stavert AR, Poulter B, Bousquet P, Canadell JG, Jackson RB, Raymond PA, Dlugokencky EJ, Houweling S, Patra PK, Ciais P, Arora VK, Bastviken D, Bergamaschi P, Blake DR, Brailsford G, Bruhwiler L, Carlson KM, Carrol M, Castaldi S, Chandra N, Crevoisier C, Crill PM, Covey K, Curry CL, Etiope G, Frankenberg C, Gedney N, Hegglin MI, Höglund-Isaksson L, Hugelius G, Ishizawa M, Ito A, Janssens-Maenhout G, Jensen KM, Joos F, Kleinen T, Krummel PB, Langenfelds RL, Laruelle GG, Liu L, MacHida T, Maksyutov S, McDonald KC, McNorton J, Miller PA, Melton JR, Morino I, Müller J, Murguia-Flores F, Naik V, Niwa Y, Noce S, O’Doherty S, Parker RJ, Peng C, Peng S, Peters GP, Prigent C, Prinn R, Ramonet M, Regnier P, Riley WJ, Rosentreter JA, Segers A, Simpson IJ, Shi H, Smith SJ, Paul Steele L, Thornton BF, Tian H, Tohjima Y, Tubiello FN, Tsuruta A, Viovy N, Voulgarakis A, Weber TS, Van Weele M, Van Der Werf GR, Weiss RF, Worthy D, Wunch D, Yin Y, Yoshida Y, Zhang W, Zhang Z, Zhao Y, Zheng B, Zhu Q, Zhu Q, Zhuang Q (2020). The global methane budget 2000–2017. Earth Syst Sci Data.

[CR35] Sekhar PK, Kysar J, Brosha EL, Kreller CR (2016). Development and testing of an electrochemical methane sensor. Sensors Actuators B Chem.

[CR36] Semrau JD, DiSpirito AA (2019). Methanotrophy – environmental, industrial and medical applications. Curr Issues Mol Biol.

[CR37] Shukla PN, Pandey KD, Mishra VK (2013). Environmental determinants of soil methane oxidation and methanotrophs. Crit Rev Environ Sci Technol.

[CR38] Sirajuddin S, Rosenzweig AC (2015). Enzymatic oxidation of methane. Biochemistry.

[CR39] Strong PJ, Xie S, Clarke WP (2015). Methane as a resource: can the methanotrophs add value?. Environ Sci Technol.

[CR40] Thauer RK (2019). Methyl (alkyl)-coenzyme m reductases: nickel F-430-containing enzymes involved in anaerobic methane formation and in anaerobic oxidation of methane or of short chain alkanes. Biochemistry.

[CR41] Trotsenko YA, Khmelenina VN (2002). Biology of extremophilic and extremotolerant methanotrophs. Arch Microbiol.

[CR42] Vivaldi F, Santalucia D, Poma N, Bonini A, Salvo P, Del Noce L, Melai B, Kirchhain A, Kolivoška V, Sokolová R, Hromadová M, Di Francesco F (2020) A voltammetric pH sensor for food and biological matrices. Sensors Actuators, B Chem 322. 10.1016/j.snb.2020.128650

[CR43] Wen G, Zheng J, Zhao C, Shuang S, Dong C, Choi MMF (2008). A microbial biosensing system for monitoring methane. Enzyme Microb Technol.

[CR44] Zhang L, Tian H, Shi H, Pan S, Chang J, Dangal SRS, Qin X, Wang S, Tubiello FN, Canadell JG, Jackson RB (2022). A 130-year global inventory of methane emissions from livestock: trends, patterns, and drivers. Glob Chang Biol.

[CR45] Zhao CG, Zheng J, Li HP, Wen GM, He YY, Yang SP, Dong C, Choi MMF (2009). Characterization of a methane-utilizing strain and its application for monitoring methane. J Appl Microbiol.

